# Improved breast cancer survival following introduction of an organized mammography screening program among both screened and unscreened women: a population-based cohort study

**DOI:** 10.1186/bcr2331

**Published:** 2009-07-06

**Authors:** Mette Kalager, Tor Haldorsen, Michael Bretthauer, Geir Hoff, Steinar O Thoresen, Hans-Olov Adami

**Affiliations:** 1 The Cancer Registry of Norway, P.O.B 5313 Majorstuen, N-0304 Oslo, Norway; 2 Department of Medical Epidemiology and Biostatistics, Karolinska Institutet, P.O.B 281, SE-17 177 Stockholm, Sweden; 3 Department of Epidemiology, Harvard School of Public Health, 677 Huntington Avenue, Boston, MA 02115, USA

## Abstract

**Introduction:**

Mammography screening reduces breast cancer mortality through earlier diagnosis but may convey further benefit if screening is associated with optimized treatment through multidisciplinary medical care. In Norway, a national mammography screening program was introduced among women aged 50 to 69 years during 1995/6 to 2004. Also during this time, multidisciplinary breast cancer care units were implemented.

**Methods:**

We constructed three cohorts of breast cancer patients: 1) the pre-program group comprising women diagnosed and treated before mammography screening began in their county of residence, 2) the post-program group comprising women diagnosed and treated through multidisciplinary breast cancer care units in their county but before they had been invited to mammography screening; and 3) the screening group comprising women diagnosed and treated after invitation to screening. We calculated Kaplan-Meier plots and multivariable Cox proportional hazard models.

**Results:**

We studied 41,833 women with breast cancer. The nine-year breast cancer-specific survival rate was 0.66 (95%CI: 0.65 to 0.67) in the pre-program group; 0.72 (95%CI: 0.70 to 0.74) in the post-program group; and 0.84 (95%CI: 0.80 to 0.88) in the screening group. In multivariable analyses, the risk of death from breast cancer was 14% lower in the post-program group than in the pre-program group (hazard ratio 0.86; (95%CI: 0.78 to 0.95, *P *= 0.003)).

**Conclusions:**

After nine years follow-up, at least 33% of the improved survival is attributable to improved breast cancer management through multidisciplinary medical care.

## Introduction

In many Western countries, breast cancer incidence is increasing, while mortality rates remain stable or are decreasing [1]. In the US, incidence rose slightly between 1987 and 2001 and then stabilized, with some evidence of decline through 2003. In contrast, death rates from breast cancer have been falling since 1990 [2]. Two obvious factors have contributed to this success: widespread use of systemic adjuvant treatment [3-5] and earlier diagnosis due to mammography screening [6,7]. The relative contributions of these factors are likely to differ between settings, population subgroups, and time periods. However, attempts to quantify them through statistical modelling suggest that in the US, adjuvants and mammography each contribute about half to the mortality reduction [8]. A Swedish study found about an 18% reduction in mortality due to factors other than screening [9].

Formation of dedicated multidisciplinary breast cancer care teams has become widespread but the potential increase in survival from this approach is not well understood. Indeed, the quantification of such a possible benefit is methodologically challenging, because it requires access to valid information on relevant confounding factors and opportunities for complete long-term follow-up of large cohorts of patients. The unique health care system, the staggered implementation of the screening program, and complete coverage of the breast cancer registry in Norway allow us to overcome such challenges.

## Materials and methods

### Norwegian health care system and the Cancer Registry of Norway

To allow all citizens equal access to public health care, charges for public medical services in Norway are kept low. As there is hardly any private in-patient service in Norway, hospital-provided medical services are population-based and mostly pertain to the county in which the patient lives. Patients rarely choose hospitals outside their county of residence, and there is no private care for breast cancer treatment [10].

Since 1952, there has been compulsory reporting of all cancer diagnoses to the nationwide Cancer Registry of Norway. Patients are referred to by their unique national registration number, which is assigned to all residents in Norway and includes the date of birth. The Registry also records information on age at diagnosis, date of diagnosis, and pathological Tumor Noduli Metastasis classification coded, according to current International Union Against Cancer guidelines [11]. Stage is coded in the Registry as I to IV: I = localized cancer, II = regional cancer, III = cancer fixed to either skin or the chest wall, and IV = cancers with distant metastases. The primary treatment is recorded as the type of surgical procedure performed. Information on primary adjuvant hormone therapy, chemotherapy, and radiation therapy is reported once by the treating surgeon and recorded as 'given' or 'not given'. Axillary surgery (axillary dissection or sentinel-node dissection) was not recorded in the database until 1993.

### Mammography screening program

**Figure 1:**
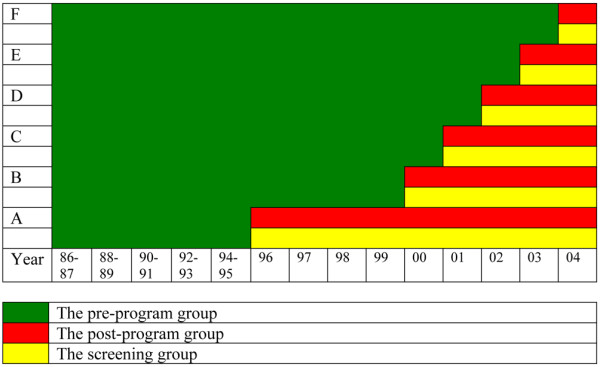
The staggered implementation of the Norwegian breast cancer screening program in the different groups of counties during the study period. The three study groups are shown in different colours. A = The counties of Rogaland, Oslo, Hordaland, and Akershus; B = Telemark, Agder, Troms, and Finnmark; C = Østfold, Nordland, Buskerud, and Trøndelag; D = Oppland and Møre og Romsdal; E = Sogn og Fjordane and Hedmark; F = Vestfold.

Following an official report that recommended a nationwide mammography screening program in Norway [12], a pilot project was started in November 1995. The plan was to invite all women aged 50 to 69 years living in four out of the 19 counties in Norway to undergo biannual mammography screening. These four counties comprised 40% of the eligible female population. After two years, the Norwegian government decided on a stepwise expansion to reach national coverage. Thus, in each county, multidisciplinary breast cancer care units were established that met the criteria of the *Quality Assurance Manual of the Norwegian Breast Cancer Screening Program *[13] based on the European guidelines for quality assurance in breast cancer screening and diagnosis [14]. To ensure high-quality medical care, as defined by the national requirements outlined in the quality assurance manual, each breast cancer care unit is continuously monitored by the Cancer Registry, including site visits every second year. During 1995 to 2004, all Norwegian counties were gradually included in the mammography screening program (Figure 1). Since February 2004, the program has been fully implemented, and all women between the ages of 50 and 69 years living in Norway are invited to undergo screening.

The national program is organized with 26 stationary and four mobile mammography screening units [13]. Women eligible for screening are identified by the Central Population Registry of Norway using their national registration number. Personal invitations are mailed to each eligible woman, suggesting an appointment time. One reminder is mailed to non-attendees [15]. Two-view mammograms, offered biannually, are read independently by two radiologists in accordance with the European guidelines for quality assurance [14]. Data on all women eligible for the screening program are stored in a central database at the Cancer Registry of Norway.

Prior to inclusion in the breast cancer screening program, each county was required to establish a multidisciplinary breast cancer care unit in accordance with the European guidelines [14] to ensure uniform management of disease. As a result, diagnosis and treatment became centralized within the county, and only dedicated teams were involved in patient care. Each breast cancer care unit covers a total population of 100,000 to 500,000. These units consist of multidisciplinary teams of radiologists, radiographers, pathologists, surgeons, oncologists, and nurses in charge of diagnosis, staging, treatment, and post-treatment surveillance of breast cancer patients. The teams meet weekly to discuss the management of newly diagnosed patients in their county. Prior to the establishment of these breast cancer care units, patients were diagnosed and treated by available radiologists, pathologists, surgeons, and oncologists, with no organized meetings or discussion of patients.

### Study cohorts

We retrieved data on all women diagnosed with a first invasive primary breast cancer in Norway between 1 November, 1985 and 31 December, 2004 from the Cancer Registry database. We achieved complete follow-up through 2004 by means of record linkages based on the national registration number. Information from the Central Population Registry allowed censoring for those individuals who emigrated. For deceased individuals, the National Death Registry provided information on date and cause of death.

We defined three mutually exclusive cohorts of women with breast cancer based on their county of residence, information on the date of the first invitation to mammography screening, and the date of diagnosis of the primary breast cancer.

*The pre-program group *comprises all women diagnosed before the mammography screening program began in their county of residence (November 1985 to February 2004).

*The post-program group *comprises all women resident in the counties where breast cancer care units were established and the screening program had started, but who had not yet been invited to mammography screening and whose breast cancer was diagnosed between November 1995 and December 2004.

*The screening group *comprises women who were diagnosed with breast cancer after they had been invited to mammography screening.

Thus, the only difference between the first two groups is that women in the post-program group received medical care through the breast cancer care units, while women in the pre-program group did not. Because the screening group is influenced by lead-time bias and length-biased sampling, their survival estimates are not directly comparable with those of the pre-program and the post-program groups, where as the latter two groups both comprise clinically detected breast cancer. As a corollary, our main comparisons were confined to the pre- and post-program groups. However, the screening group was included to allow a conservative estimate of the proportional contribution of improved management as distinct from screening detection.

### Statistical analyses

We used the Pearson chi-squared test to compare the two groups according to age, stage of breast cancer, and treatment. The overall and breast cancer-specific survival rates were calculated using life table techniques, illustrated by Kaplan-Meier plots, and compared using the log-rank test. Censoring occurred at date of emigration, date of death from causes other than breast cancer, or the end of the follow-up period, whichever came first. To facilitate comparison, we restricted follow-up in the pre-program group in accordance with follow-up period in the post-program group. Logically, the counties most recently included in the screening program had a shorter follow-up time. We restricted follow-up time in the pre-program group to the actual follow-up time in the post-program group in each county. Hence, for the most recently enrolled county, follow-up time was 0.9 years in the post-program era, and we restricted follow-up time in the pre-program group accordingly. Time trends of breast cancer survival were calculated for each county.

Hazard ratios were calculated using Cox proportional hazard models. We used the likelihood ratio statistics to compare groups. We adjusted for age at diagnosis by four age categories: younger than 40, 40 to 49, 50 to 69, and 70 years and older. We further adjusted for time trends, county of residence, and the following treatment variables: surgery (yes/no), hormone treatment (yes/no), chemotherapy (yes/no), and radiation therapy (yes/no). Because trends in survival differed by county, we adjusted for county and time trends by including county-specific trend parameters in the model. We did not adjust for stage at diagnosis due to the likelihood of stage migration [14]. The proportional hazard assumption was tested by both graphical methods and Schoenfeld residuals.

All test statistics are two-tailed and *P *values < 0.05 are considered statistically significant. All calculations are performed with the statistical package Stata 9.2 (StataCorp, Tx, USA). The research protocol was approved by the ethical review board of the Cancer Registry of Norway. Individual informed consent was not requested.

## Results

### Baseline characteristics

We identified a total of 41,833 women diagnosed with a primary invasive breast cancer from 1 November, 1985 to 31 December, 2004. During follow-up through 2004, 16,494 (39.4%) women died, for 9953 (60.3%) of whom breast cancer was listed as the underlying cause of death. A total of 6960 women were diagnosed after they had been invited to the screening program. This left a total of 34,873 women to be included in the main comparisons between the pre- and post-program group. Of these, 26,883 (77.1%) individuals belong to the pre-program group, while 7990 (22.9%) comprise the post-program group. The mean follow-up times in the pre- and post-program groups were 3.8 years and 3.2 years, respectively. The maximum duration of follow-up was 9.1 years in both groups.

Table 1 shows baseline characteristics for the pre-program and post-program groups of breast cancer patients. The proportion between 50 and 69 years of age was smaller in the post-program group than in the pre-program group because only women in that age group are invited to mammography screening. Thus, these patients were included in the screening group. Compared with the pre-program period, more patients in the post-program era received tamoxifen and radiation therapy. In addition, chemotherapy was given more often to women younger than 50 years than to those aged over 50 years in the post-program period. Approximately 10% of all patients had no surgery; most of these women were older than 69 years (Table 1).

**Table 1:** Baseline characteristics of women with incident breast cancer in Norway 1985 to 2004, diagnosed before (pre-program) and after (post-program) introduction of population-based mammography screening program in their county of residence

	**Pre-program** **n = 26,883**		**Post-program** **n = 7990**	
**Characteristic**	**Number**	**(%)**	**Number**	**(%)**
Age (years 0)				
<40	1492	5.6	658	8.2
40 to 49	4533	16.9	2119	26.5
50 to 69	10,339	38.5	1383	17.3
≥ 70	10.519	39.1	3830	47.9
Stage*				
I	13,035	48.5	3977	50.0
II	10,338	38.5	3201	40.1
III	1414	5.3	348	4.4
IV	1750	6.5	444	5.6
unknown^#^	346	1.3	20	0.3
Pathologic size				
1 (0 to 20 mm)	7782	28.9	3162	39.6
2 (>20 to 50 mm)	5657	21.0	2270	28.4
3 (>50 mm)	621	2.3	190	2.4
4 (growth)	1429	5.3	443	5.5
unknown^#^	11,394	42.4	1925	24.1
Treatment^‡^				
Radiation therapy	5323	19.8	2942	36.8
unknown^#^	5113	19.0	1847	23.1
Hormone therapy	8230	30.6	3128	39.2
unknown^#^	4810	17.9	2041	25.5
Chemotherapy	6792	25.3	2044	25.6
unknown^#^	5523	20.5	2678	33.5
Surgery	24,694	91.9	6997	87.6
BCT	2160	8.0	2045	25.6
Mastectomy	20,661	76.9	4885	61.1
SN**	572	4.2	1859	23.3
Ax**	10,356	75.6	4486	56.2

^#^unknown represents either missing values or values we were not able to classify.

* staging is based on the classification of tumor-stage in the Cancer Registry of Norway: I = localized cancer, II = regional cancer (represents no of node 0 positive tumors), III = cancer fixed to either skin or to the chest wall, IV = cancers with distant metastases.

^‡^the treatment variable indicates that treatment was given.

**surgical procedures in the axilla were not specified until 1993 in the database; percentage is calculated based on breast cancer patients diagnosed from 1993 to 2004.

Ax = axillary dissection; BCT = breast-conserving surgery; SN = sentinel-node dissection.

### Survival analyses

**Figure 2:**
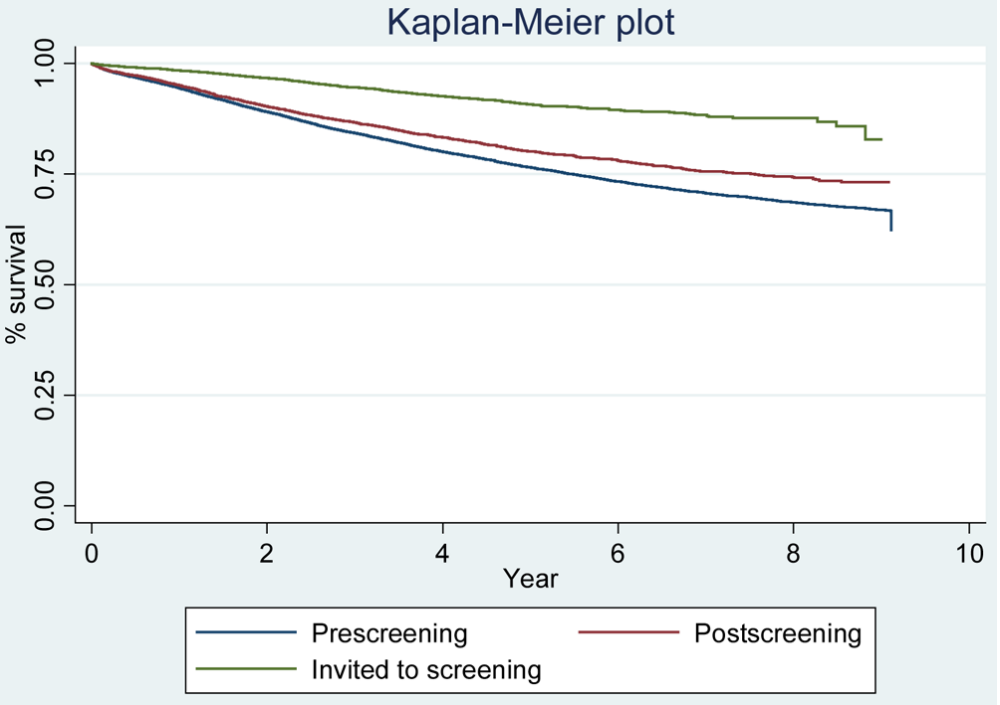
Survival curves for the different groups.

**Figure 3:**
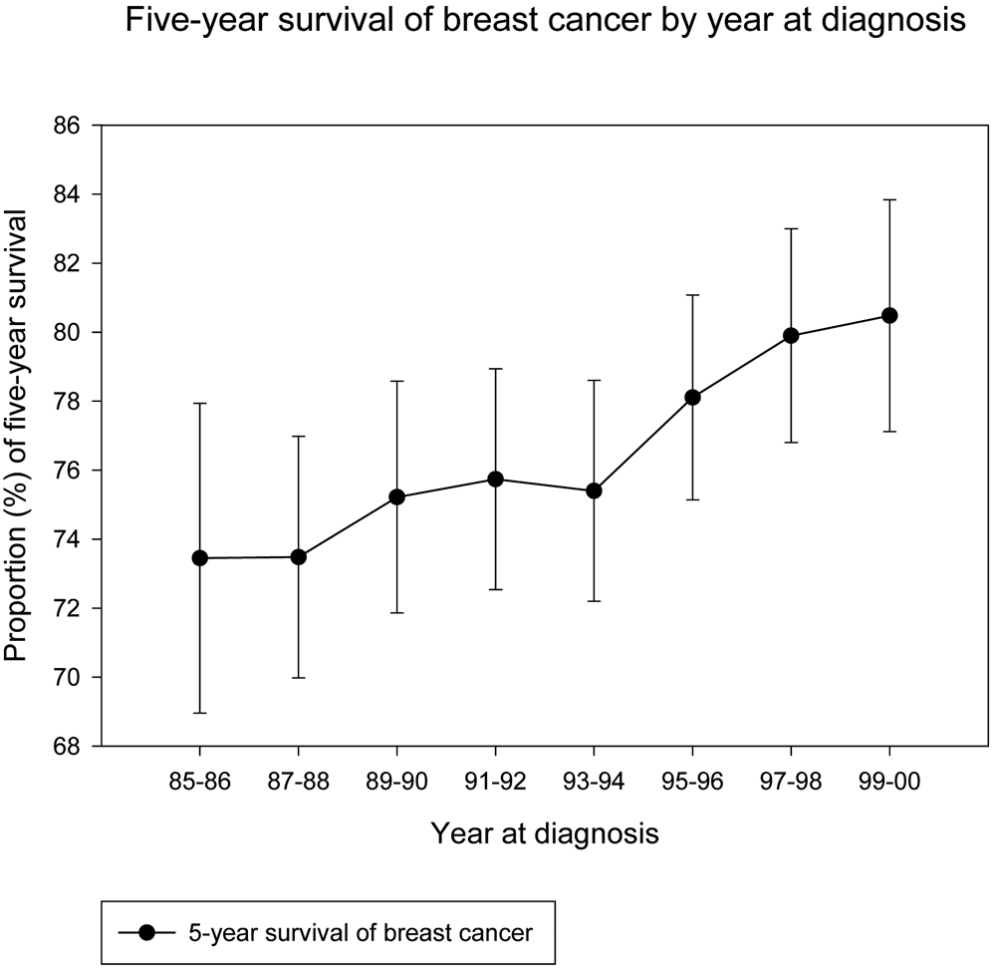
Five-year survival of breast cancer by two-year increments throughout the study period for all women except those invited to mammography screening. The curve is presented with 95% confidence interval.

Breast cancer survival rates were significantly different across the three cohorts (*P *< 0.001; Figure 2). Compared with the pre-program group, estimated nine-year survival was 6% higher in the post-program group and 18% higher among women who had been invited to mammography screening (Table 2). In exploratory analyses stratified by stage of disease, the five- and nine-year survival was consistently higher in the post-program than in the pre-program group (data not shown). Five-year survival for women diagnosed in successive two-year intervals increased gradually with a more marked improvement following introduction of mammography screening program in 1995 to 1996 (Figure 3).

**Table 2:** Five- and nine-year breast cancer-specific survival in the pre-and post-program group and screening group

	**5-year**		**9-year**	
	
**Group**	**Survival**	**95% CI**	**Survival**	**95% CI**
**Pre-program group**	0.76	0.75 to 0.77	0.66	0.65 to 0.67
**Post-program group**	0.80	0.79 to 0.81	0.72	0.70 to 0.74
**Screening group**	0.91	0.90 to 0.92	0.84	0.80 to 0.88

CI = confidence interval.

Compared with the pre-program period, women diagnosed in the post-program period experienced a statistically significant 14% reduction in breast cancer-specific death rate after adjustment for age, calendar year of diagnosis, county of residence, and treatment variables (hazard ratio (HR) 0.86; 95% confidence interval (CI) 0.78 to 0.95; *P *= 0.003; Table 3). An additional analysis included only those individuals in whom all treatment variables were available (n = 23,111), with no significant difference in HR compared with the main analysis (HR 0.83; 95% CI 0.72 to 0.95; *P *= 0.009). In an additional analysis of only the four pilot counties, we noted a 19% reduction in breast cancer-specific death rate when adjusting for age, calendar year of diagnosis, county of residence, and treatment variables (HR 0.81; 95% CI 0.71 to 0.91; *P *= 0.001). In analyses without adjusting for calendar year of diagnosis, we found a 24% reduction (HR 0.76; 95% CI 0.70 to 0.82; *P *< 0.001).

**Table 3:** Hazards ratios from Cox regression models with 95% confidence interval and corresponding *P *values, of breast cancer survival comparing post-program group with pre-program group

	**Univariable**			**Multivariable**		
		
Category	**HR 95% CI**		***P *value**	HR* 95% CI		***P *value**
**Pre-program**	1.00	ref		1.00	Ref	
**Post-program**	0.83	0.77 to 0.88	<0.001	0.86	0.78 to 0.95	0.003
						
**Age-categorized (years)**						
0 to 39				1.00	ref	
40 to 49				0.60	0.53 to 0.67	<0.001
50 to 69				0.73	0.66 to 0.81	<0.001
≥ 70				0.98	0.89 to 1.08	0.75
Surgery performed^#^				0.17	0.16 to 0.19	<0.001
Chemotherapy given^#^				0.97	0.96 to 0.98	<0.001
Hormone therapy given^#^				1.03	1.01 to 1.04	<0.001
Radiation given^#^				1.00	0.99 to 1.02	0.63

*Adjusted for county of residence and calendar year of diagnosis.

^# ^Reference categories: surgery not performed; chemotherapy not given; hormone therapy not given; radiation not given, respectively.

CI = confidence interval; HR = hazard ratio.

## Discussion

Our data show a substantial survival benefit among women diagnosed with breast cancer after introduction of the Norwegian breast cancer screening program but before they had been invited to mammography. We consider this benefit likely to be due to a combination of earlier diagnosis through increased awareness and optimization of standard breast cancer care through establishment of breast cancer care units. Thus, the implementation of the larger breast cancer screening program, including a re-organization of the breast cancer health care system, significantly increased survival independent of the primary task of the program, namely mammography screening.

The fundamental prerequisite for our analysis was the large overlap in time between the pre- and post-program group, due to the step-wise introduction of mammography screening in all Norwegian counties during a nine-year period. This overlap minimized potential confounding due to factors that might affect the survival of breast cancer in the population over time. Additional strengths of our study include its nationwide design, large size, complete long-term follow-up of patients, and the homogeneous public health care system in Norway, which offers uniform health care management to all women diagnosed with breast cancer in the same county at a certain time.

Our study has several potential limitations. We were not able to take into account mammography screening through private medical services, which might confound our survival analyses if it affects the women in pre- and post-program groups differently. Confined mostly to the larger cities of Norway, private mammography screening has been available since the mid-1980s. However, there is no evidence that it has become more common during the study period [16]. If the improvement in breast cancer survival was caused mainly by improved breast cancer awareness or private screening, we would expect smaller, less advanced cancers in the post-program group. However, the stage distribution was strikingly similar in the pre- and post-program periods (Table 1), although stage migration due to more sensitive diagnostic methods in recent years might have affected this comparison.

We saw an improvement in breast cancer survival in all stages, which might represent a real survival benefit, but may also have been influenced by stage migration [17]. In contrast to other studies [18,19], ours did not adjust for stage, because stage migration might entail differential misclassification between the pre- and post-program groups. Despite the overlap in time between the two groups, we adjusted for time trends and treatment variables, with no significant differences in treatment variables between the two groups. The adjustment for time trend may give the most reliable results in our model, as the treatment-related data at the Registry are only recorded once, and this is most often performed at the time of the primary surgery, before treatment is completed.

Furthermore, 40% of the eligible women in Norway were offered mammography screening from the start of the program in 1995/6. To explore the effect of overall improvement in breast cancer mortality during the time period 1985 to 2004, we performed separate analysis for the women living in the four pilot counties. In this comparison, the pre- and post-program groups are not parallel in time. Here, we found a 19% reduction in breast cancer mortality after adjusting for calendar year of diagnosis and a 24% reduction when we did not adjust for calendar year of diagnosis. This 5% difference might be attributable to a gradual improvement in diagnosis and treatment, enhanced breast cancer awareness, improved general health, and other factors not easily measured over the time period. We noted a gradual improvement in breast cancer mortality throughout the time period, so we chose to adjust for calendar year of diagnosis, not to exaggerate the effect of implementation of multidisciplinary breast cancer care.

It is impossible to reliably attribute the observed survival benefit to any specific component of optimal clinical management. New surgical treatments, such as breast-conserving surgery, combined with radiation therapy, sentinel node techniques, and axillary node dissection do not improve the survival rate of patients with breast cancer [20-22]. However, several studies have shown a positive association between surgical experience (measured in number of procedures performed) and survival [23-25]. The proportion of patients treated with chemotherapy across all age groups is stable throughout the study period. However, it is conceivable that such treatment was given more systematically to patients who benefited most when they were managed by multidisciplinary team work. Not surprisingly, we found that women who did not have surgery had poorer outcomes.

To the best of our knowledge, the present study provides the first empirical data on non-mammography benefits of mammography screening programs. Consistent with our findings, however, a computer-based modelling by Blanks and colleagues estimated a 15% reduction in mortality by factors beyond mammography screening in a national mammography screening program in the UK [26]. The survival curves of breast cancer patients diagnosed by screening are not directly comparable with survival curves of women diagnosed clinically. In the former category, survival time becomes exaggerated by lead-time bias, length bias sampling, and possibly also by selection bias and overdiagnosis bias. Because we are unable to adjust for screening survival biases, the proportional contribution by factors other than screening is underestimated. In addition, the proportion of women aged 50 to 69 years was higher in the screening group than the pre-screening group, which may lead to further underestimation of the difference in breast cancer survival among women in the two groups, because women diagnosed at this age have a higher breast cancer survival than women younger than 50 years and older than 69 years of age. Based on nine years of follow-up, we found that at least 33% ((0.72 to 0.66)/(0.84 to 0.66)) of the increase in survival following the introduction of the mammography screening programs is attributable to better management of breast cancer.

## Conclusions

Our results suggest an independent improvement in breast cancer survival produced by reorganizing breast cancer care, probably combined with earlier diagnosis through increasing awareness of early breast cancer symptoms. Because differences in prognosis and survival are seen for a wide variety of malignancies, between geographic areas, and among different socioeconomic classes, it is intriguing to believe that this effect may apply not only to the management of breast cancer patients, but to a variety of other malignancies as well.

## Abbreviations

CI: confidence interval; HR: hazard ratio.

## Competing interests

The authors declare that they have no competing interests.

## Authors' contributions

MK conceived and designed the study, analysed and interpreted the data, and drafted the manuscript. HOA co-designed the study, interpreted the data, and co-drafted the manuscript. TH co-designed the study, analysed and interpreted the data, and revised the manuscript. MB interpreted the data and co-drafted the manuscript. GH co-designed the study and revised the manuscript. ST co-designed the study and revised the manuscript. All authors read and approved the final version of the manuscript.
